# Dysregulated expression of hypoxia-inducible factors augments myofibroblasts differentiation in idiopathic pulmonary fibrosis

**DOI:** 10.1186/s12931-019-1100-4

**Published:** 2019-06-24

**Authors:** Arnoldo Aquino-Gálvez, Georgina González-Ávila, Laura Lorena Jiménez-Sánchez, Héctor Aquiles Maldonado-Martínez, José Cisneros, Fernanda Toscano-Marquez, Manuel Castillejos-López, Luz María Torres-Espíndola, Rafael Velázquez-Cruz, Víctor Hugo Olivera Rodríguez, Edgar Flores-Soto, Héctor Solís-Chagoyán, Carlos Cabello, Joaquín Zúñiga, Yair Romero

**Affiliations:** 1 Instituto Nacional de Enfermedades Respiratorias Ismael Cosío Villegas, Tlalpan 4502, 14080 Mexico City, CP Mexico; 2 Instituto Nacional de Cancerología, Mexico City, Mexico; 3 Facultad de Ciencias, Universidad Nacional Autónoma México, Mexico City, Mexico; 4 Instituto Nacional de Pediatría, Mexico City, Mexico; 5 Instituto Nacional de Medicina Genómica, Mexico City, Mexico; 6 Departamento de Farmacologia, Facultad de Medicina, Universidad Nacional Autónoma México, Mexico City, Mexico; 7 Instituto Nacional de Psiquiatría Ramón de la Fuente Muñiz, Mexico City, Mexico; 8 Escuela de medicina y ciencias de la salud, Tecnologico de Monterrey, Mexico City, Mexico

**Keywords:** Hypoxia inducible factors; αSMA, Lung fibroblasts, HIF-1α, HIF-2α, HIF-3α, Methylation

## Abstract

**Background:**

Idiopathic pulmonary fibrosis (IPF) is an age-related, progressive and lethal disease, whose pathogenesis is associated with fibroblasts/myofibroblasts foci that produce excessive extracellular matrix accumulation in lung parenchyma. Hypoxia has been described as a determinant factor in its development and progression. However, the role of distinct members of this pathway is not completely described.

**Methods:**

By western blot, quantitative PCR, Immunohistochemistry and Immunocitochemistry were evaluated, the expression HIF alpha subunit isoforms 1, 2 & 3 as well, as their role in myofibroblast differentiation in lung tissue and fibroblast cell lines derived from IPF patients.

**Results:**

Hypoxia signaling pathway was found very active in lungs and fibroblasts from IPF patients, as demonstrated by the abundance of alpha subunits 1 and 2, which further correlated with the increased expression of myofibroblast marker αSMA. In contrast, HIF-3α showed reduced expression associated with its promoter hypermethylation.

**Conclusions:**

This study lends further support to the involvement of hypoxia in the pathogenesis of IPF, and poses HIF-3α expression as a potential negative regulator of these phenomena.

## Background

Idiopathic Pulmonary Fibrosis (IPF) is a chronic and progressive lung disorder of unknown etiology. IPF typically occurs in persons over sixty years old. It is characterized by alveolar epithelial injury followed by expansion of the fibroblast/myofibroblast population and exaggerated accumulation of extracellular matrix (ECM), all of which result in the loss of lung function [1–3].

The molecular mechanisms sensing oxygen concentrations are required to establish a healthy physiological state. In this study, the focus was on hypoxia-inducible alpha subunits. However, the response to hypoxia occurs in several molecular mechanisms, this system nevertheless appears to have a critical role in the lung homeostasis and the pathogenesis of IPF [4]. Hypoxia-inducible factors (HIFs) are able to exponentially increase their transcriptional activity in response to oxygen availability decline. These transcriptional factors are heterodimeric composed of an alpha and a beta subunit, each with basic helix-loop-helix (bHLH) domain and PAS (Per-AHR-ARNT-Sim) domain. The levels of alpha subunits in normoxia (−1α, −2α, −3α) are restricted by ubiquitination and proteasomal degradation in control of oxygen-dependent prolyl hydroxylation and von Hippel–Lindau (VHL) protein recognition [5].

Several studies have demonstrated a direct link between hypoxia and the development of IPF, principally by the notion that hypoxia worsens by constant deposition of ECM and vice versa [6–8]. Whole lung transcriptome of IPF patients has shown that hypoxia signaling pathway is upregulated and denotes the signature of HIF-1α [5, 6]. At cellular level hypoxia signaling by HIF-1alpha is able to modify the behavior of IPF derived fibroblasts increasing their proliferation [9, 10]. Therefore, the scarring of lung tissue in IPF is reinforced by the expansion of fibroblast/myofibroblasts foci. However, the correlation between fibroblast foci formation and hypoxia signaling is not well described, and we can now provide additional insights into this process.

## Methods

### Human lung fibroblasts

The study was approved by the ethics committee of the Instituto Nacional de Enfermedades Respiratorias (INER). Written informed consent was obtained from all participating individuals. Human fibroblasts cell lines were obtained by surgery (*n* = 4) and in the case of controls were taken from heathy lungs (*n* = 4) that were not suitable for transplant. IPF diagnosis depends on the department of interstitial lung diseases of the INER according to the ATS/ ERS/ ALAT guidelines [11, 12]. Approximately 20% of patients requiring surgery biopsy for definitive diagnostic, and this decision is established in an MDD (multi-disciplinary board) when tomographic criteria or clinical data are unclear. So this group is used in this study once the final diagnosis for IPF is confirmed (90% confidence). Fibroblasts were derived from lung tissue obtained from IPF patients and controls were isolated by enzymatic dispersion with trypsin (Sigma-Aldrich). Cells were grown with Ham’s F-12 medium (Gibco) supplemented with 100 U/mL of penicillin, 100 μg/ml of streptomycin, 2.5 mg/ml of amphotericin B and 10% FBS (Gibco); at 37 °C; in an atmosphere of 95% air and 5% CO2; until reaching early confluence. All experiments were performed on cells with passage number between 5 to 10.

### Hypoxia

For studying hypoxic conditions, fibroblast culture plates were transferred and maintained in a modular incubator chamber (Billups-Rothenberg Inc. CA.), at 37 °C, for different periods of time, according to the experiment, in a humidified atmosphere with the following hypoxic gas mixture: 1% O2, 5% CO2, and balanced with N2. An Oxygen Analyzer (Teledyne Electronic Technologies 60 T) with an Oxygen Sensor (OOM105 of EnviteC-Wismar GmbH) to regulate the atmosphere composition.

### Western blot

Cells were lysed with RIPA buffer (Sigma- Aldrich, St. Louis, MO). Protein quantification was performed using the BCA Protein Assay Kit (Thermo Scientific, USA). 30 μg from total extracts were separated by 10% SDS polyacrylamide gels (PAGE), and proteins were then transferred to nitrocellulose membranes. After nonspecific sites blockaded with 4% (wt/vol) of non-fat dried milk in phosphate-buffered saline (PBS), the membrane was incubated overnight with the correspondent antibody: HIF-1α (Abcam; ab1), HIF-2α (Novus; NB100–122) and HIF-3α (Novus; NBP1–03155), and αSMA (Sigma; A2547). β-actin (Sigma, A5441) was used as housekeeping. Bands were detected using secondary antibodies (Licor) and Odyssey scanner (Licor) detection system. Quantification was performed using ImageJ software (NIH).

### Quantitative PCR

RNA was extracted using TRIzol reagent (Invitrogen Life Technologies, Grand Island, NY). One microgram of total RNA was used to generate cDNA (Advantage RT-for-PCR Kit; Clontech, Palo Alto, CA). Real-time PCR was carried out on a StepOne Thermocycler (Applied Biosystems, Foster City, CA) using Applied Biosystems TaqMan® Gene Expression Master Mix (Applied Biosystems, Foster City, CA.) according to the manufacturer’s protocol. The expression assay was carried out using the following TaqMan probes: Hs00153153_m1 for HIF-1α, Hs01026149_m1 for HIF-2α (EPAS1), Hs00541709_m1 for HIF-3α, Hs00426835_g1 for ACTA2 (αSMA) all of the above labeled with FAM and normalized with Hs99999901_s1 for 18S ribosomal RNA labeled with VIC (Applied Biosystems, Foster City, CA). Relative quantitation method was used to analyze the results of two independent experiments made in triplicate. For each experimental sample, a gene was considered as not expressed if amplification was not detected by threshold cycle Ct = 40.

### Immunohistochemistry on tissue microarrays and Immunocitochemistry

For lung samples tissue analysis, tissue microarrays (TMA) were constructed using paraffin blocks from the pathology archive. Samples for IPF (*n* = 10), other inflammatory diseases (*n* = 5), from lung cancer patients (*n* = 2), and normal residual lung tissue from patients with cancer (*n* = 7). The tissue microarray was constructed using one core of 5 mm in diameter per sample.

Positive control tissue from normal kidney and placenta were used for optimal antibody titration, before immunohistochemistry reactions were performed on tissue microarray or cellular culture slides. All slides were prepared by placing positive and negative control tissue on them. Primary antibody specifications are shown in Table 1. For TMA paraffin sections the reactions were performed manually as follows: 1) Slide deparaffinization at room temperature in successive incubations in xylene, absolute ethanol and 96° ethanol, 2) Antigen retrieval with citrate buffer (pH 6, pressure cooker) for 10 min at 95°c for HIF-1α, HIF-2α and αSMA; and with 1x proteinase K (Diagnostic Biosystems, Pleasanton, CA) for 20 min at room temperature for HIF-3α; 3) Peroxidase blockade, using a 3% solution of hydrogen peroxide in methanol, for 10 min at room temperature; 4) Primary antibody incubation overnight in a humidity chamber according to Table 1 titers; and 5) Polymer-horseradish peroxidase based detection with diaminobenzidine as chromogen (MACH 4, Biocare Medical, Pacheco, CA). In between all steps, thorough washings were made with Tween 20 – PBS. All slides were counterstained with Harris Haematoxylin and mounted with non-aqueous medium. For immunocytochemical reactions on slide cell cultures of fibroblasts lines, the procedure was similar to TMA paraffin sections, just substituting the initial deparaffinization steps for a membrane permeabilization step through: incubation in 70% methanol for 1 h. TMA immunohistochemistry reactions for HIF-1α, HIF-2α and HIF-3α were evaluated by a pathologist in fibroblasts, lymphocytes, macrophages, plasma cells and alveolar epithelium. Percentage of positive cells and reaction intensity were registered as categorical data according to the next ranges: A) Percentage.- 0 for 0% of positive cells, 1 for < 10% of positive cells, 2 for 10–50% of positive cells and 3 for > 50% of positive cells; and B) Intensity.- 0 for negative reactions, I for light staining (barely perceptible under 10x objective, but well defined stain visible under 40x objective), II for moderate staining (well defined not strong stain under 10x objective), and III for strong staining (evident intense stain even under 4x objective).

**Table 1:** List of primary antibodies

Target	Antibody features	Trademark	Titer
HIF1α	Mouse monoclonal	Abcam (ab 1)	1:50
HIF2α	Rabbit polyclonal	Novus (NB100-122)	1:150
HIF3α	Rabbit polyclonal	Novus (NBP1-03155)	1:150
ACTA	Mouse Monoclonal	Sigma-Aldrich (A2547)	1:1000

### Demethylation test (5-Aza)

IPF fibroblasts were seeded in a 6-well plate at 30% confluence in complete F12 medium and were treated with 5-Aza-2′-deoxycytidine (5-Aza) (A3656 Sigma) for 5 days, the medium was changed every 24 h. After the treatment with the inhibitor, the cells were lysed.

### Statistical analysis

Before each statistical analysis of quantitative variables, a Shapiro-Wilk test was performed to evaluate normality distribution of the data. For western blot and genic expression analysis, the paired t-test was used for intra-group comparisons of each sample or group studied. Inter-group comparisons were performed using the Levene’s test and independent t-tests, Tukey tests, to investigate the differences between groups. All statistical analyses were performed with the IBM SPSS Statistics 20.0 (Chicago, Illinois, USA) software. *P* < 0.05 was considered statistically significant. For immunohistochemistry, results were compared between normal control tissue samples with either idiopathic pulmonary fibrosis or other pulmonary inflammatory conditions. 4 × 2 contingency tables were constructed, and the Freeman-Halton extension of the Fisher exact probability test performed on them.

## Results

### Hypoxia contributes to myofibroblast differentiation in IPF

**Fig. 1:**
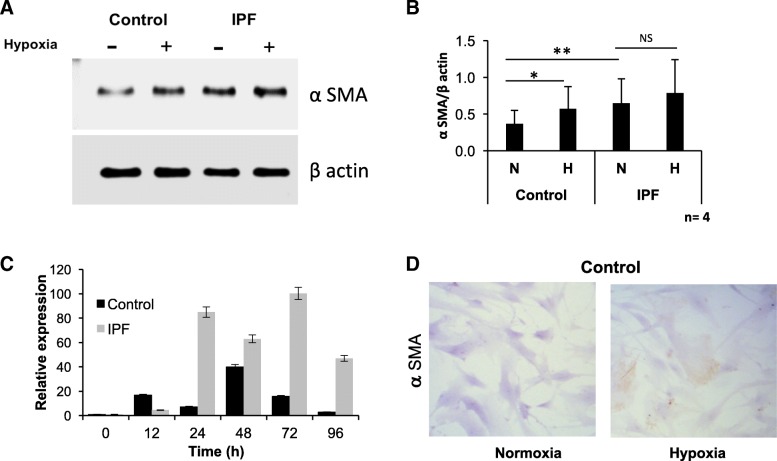
Fibroblasts have increased αSMA expression in IPF and hypoxia exposure**.** Representative western blots of αSMA (**a**) after 48 h of exposure to hypoxia (1% O2). β-actin was used as a loading control. **b** Each bar represents the mean ± SD of the 4 fibroblast lines for each group. *P < 0.05, **P < 0.01 two-tailed Student’s t-test. Gene expression of αSMA (**c**) at basal (0 h) versus 12, 24, 48, 72 and 96 h of exposure to hypoxia (1% O2) of the two fibroblast lines. The results are expressed in arbitrary units. Representative immunocytochemistry of normal fibroblast lines after 48 h of exposure to hypoxia (**d**)

αSMA expression was evaluated as a marker of myofibroblast differentiation which is a key feature in the pathogenesis of IPF. To determine expression levels of αSMA, four lines of fibroblasts controls were obtained with an average age similar to the lines derived from patients with IPF, all of them were exposed for 48 h to 1% Oxygen. Control fibroblasts showed a significant increase in the protein expression after hypoxia (*p* = 0.01) (Fig. 1a-b). As it can be also see by immunocytochemistry in (Fig. 1d). IPF derived fibroblasts exhibited a significant difference in baseline conditions compared to controls (*p* = 0.008); however, after the hypoxic stimulus, the increase is marginal in comparison to baseline (*p* = 0.19) (Fig. 1a-b). αSMA gene (ACTA2) expression increases in control fibroblasts at 12, 24, 48, 72 and 96 h, with a peak after 48 h which returns to baseline level at 96 h (Fig. 1c). In IPF cells, αSMA expression is higher than in controls and the level remains high even after 96 h (Fig. 1c) (Table 2). These results indicate that hypoxia induces myofibroblast differentiation, this phenotype is still present in conditions with available oxygen in IPF.

**Table 2:** *P*-values of gene expression (hypoxia vs basal conditions)

Gene	12	24	48	72	96hrs
Control					
HIF1	0.032	0.047	0.002	<0,001	0.040
HIF2	0.011	<0.001	<0.001	<0,001	<0,001
ACTA	0.001	0.001	<0.007	0.002	<0,001
IPF					
HIF1	0.010	<0.001	<0.001	<0,001	0.136
HIF2	0.037	<0.001	0.002	<0,001	0.004
ACTA	0.001	<0.001	<0.001	<0,001	<0,001

### Active hypoxia signaling in IPF derived fibroblasts by HIF-1α & HIF-2α but no HIF -3α

**Fig. 2:**
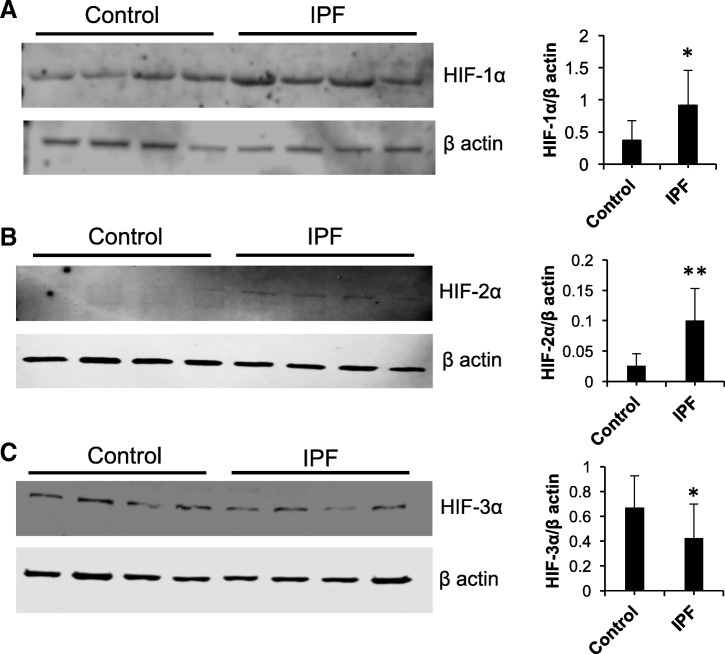
Basal expression of hypoxia-inducible alpha subunits on IPF derived fibroblasts. Western blots of HIF1α (**a**), HIF2α (**b**) & HIF3α (**c**) in fibroblasts from IPF lungs and controls cultured at 37 °C in an atmosphere of 95% air and 5% CO2, passage 5–7. β-actin was used as a loading control. Densitometric analysis, each bar represents the mean ± SD of 4 different cell lines for each group. *P < 0.05, **P < 0.01 two-tailed Student’s t-test

For decades, reports have established that hypoxic condition is associated with the production and accumulation of ECM molecules in fibroblasts of different tissues [13–18]. To evaluate whether hypoxia signaling may be a factor in ameliorating the differentiation process, HIFs were measured. The basal levels of proteins HIF-1α, HIF-2α and HIF-3α showed statistically significant differences, as it is shown in Fig. 2. HIF-1α is increased in IPF (*p* = 0.05) (Fig. 2 a) as well as HIF-2α (*p* = 0.004) (Fig. 2 b), while HIF-3α presents a different behavior with more HIF-3α protein in controls (*p* = 0.04) (Fig. 2 c). These results correlate with the differences in the expression of αSMA in basal conditions because HIF-1α and -2α participate in the activation of hypoxia signaling while HIF-3α has been reported that regulates this pathway negatively [19].

**Fig. 3:**
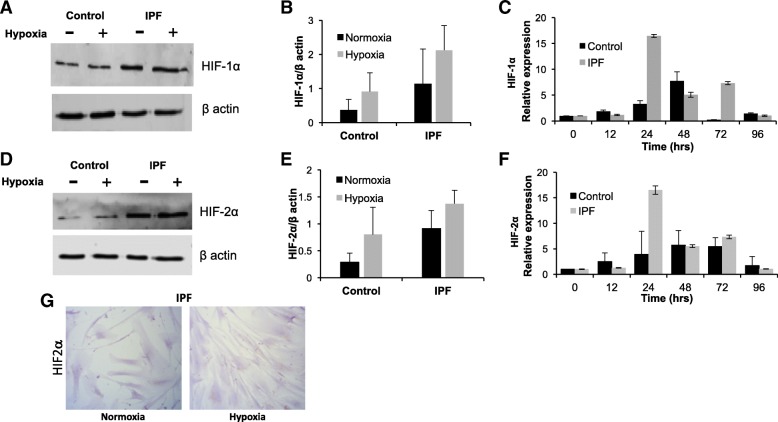
IPF derived fibroblasts have high levels of HIF-1α & -2α expression. Representative western blots of HIF1α (**a**) and HIF2α (**d**) after 48 h of exposure to hypoxia (1% O2). β-actin was used as a loading control. Each bar represents the mean ± SD of the two fibroblast lines for each group (**b**, **e**). Gene expression of HIF1α (**c**) and HIF2α (**f**) at basal (0 h) versus 12, 24, 48, 72 and 96 h of exposure to hypoxia (1% O2) of the normal fibroblast and in fibroblast from idiopathic pulmonary fibrosis. Representative immunocytochemistry of HIF2α in IPF fibroblast and in normoxia and after 48 h of exposure to hypoxia (**g**)

In addition, it was evaluated the dynamics of the response and it was found that when controls are exposed to hypoxia for 48 h, these cells increase their amount of HIF-1α and HIF-2α proteins with respect to normoxia (Fig. 3 a, b, d, e). However, in IPF, the amount of these two proteins showed a slightly increase (Fig. 3 a, d). Therefore, the levels of these proteins come from a previous stimulus and remain in cells derived from IPF.

By qPCR, gene expression in control fibroblasts displays, after 12, 24, 48, 72 and 96 h of exposure to hypoxia (1% O2), a peak after 48 h for HIF-1α and at 72 for HIF-2α (EPAS1) (Fig. 3 c, f). IPF cells present a peak after 24 h in both (Fig. 3 c, f). Table 2 shows statistical differences between each different time slot and baseline (0 h). In all cases, there was a significantly increased expression of the genes in hypoxic fibroblasts when compared to basal either for controls or for IPF (*p* < 0.05). Table 3 indicates intergroup comparisons (normal vs IPF), which are statistically significant for HIF-1 and HIF2 (EPAS1) at 12, 24 and 72 h. In most cases, IPF cells show higher levels in response to hypoxia and remain longer. Immunocytochemistry in fibroblast supports the findings for the expression of HIF-2α (Fig. 3 g). Summarizing the results, there was an increased expression of hypoxia signaling with higher levels for IPF cell lines, mainly by HIF-2α.

**Table 3:** P-values of gene expression after hypoxia exposure (IPF vs control)

Gene	12	24	48	72	96 hrs
HIF1	0.025	<0,001	0.006	<0,001	0.046
HIF2	0.012	<0,001	0.018	<0,001	0.114
ACTA	0.005	<0,001	<0,001	<0,001	<0,001

### HIF-3α hypermethylation could be responsible for augment myofibroblasts differentiation in IPF

**Fig. 4:**
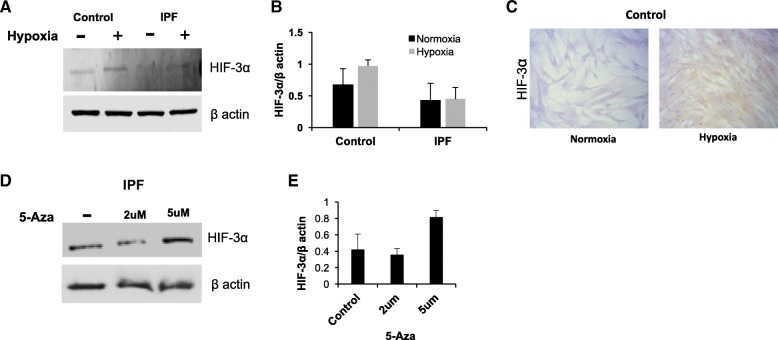
IPF derived fibroblasts have low expression of HIF-3α by hypermethylation. Representative western blots of HIF3α (**a**) after 48 h of exposure to hypoxia (1% O2). β-actin was used as a loading control. Each bar represents the mean ± SD of the two fibroblast lines (**b**). Representative immunocytochemistry of HIF3α in normal fibroblast in normoxia and after 48 h of exposure to hypoxia (**c**). Western blot of HIF3α in IPF derived fibroblasts after treatment with 2 and 5uM of 5-Aza-2′-deoxycytidine (5-Aza) for 5 days. β-actin was used as a loading control (**d**). Each bar represents the mean ± SD of the two experiments (**e**)

HIF-3α is the less studied isoform, this gene produces a large number of variants by alternative splicing [19–21]. It has been accepted that the majority of these variants act as negative feedback regulators of the activity of HIF-1α and HIF-2α [14, 18–21]. According to this, the increase in the differentiation could be as a result of significant lower levels of HIF-3α protein in IPF compare to controls (*p* = 0.04) (Fig. 2 c). The results were even more striking in hypoxia, because in the HIF-3α promoter region there are hypoxia response elements (HRE), characteristic of the genes that are targeted by HIF-1α [14–17]. In controls, it exhibits a similar way of HIF (1 and 2) proteins, these cells increase the amount of HIF-3α but in IPF their levels remain unchanged (Fig. 4a-c). Unfortunately, for HIF3a, there is no data of PCR amplification for any sample most likely due to the probe used.

Hypoxia induces global DNA hypermethylation [18, 22]. For example, in prostate cancer, HIF-3α gene has been found hypermethylated compared to healthy tissue from the same patient and this promotes carcinogenesis [23]. Following these observations, it was tested if promoter methylation could be the reason for the decrease in HIF-3α. In the demethylation assay with 5-Aza, two IPF lines without response were tested. Aza treatment increased the levels of HIF-3α protein, suggesting that the lack of hIF3alpha is due to hypermethylation (Fig. 4d, e).

### Integrated evaluation of hypoxia-regulated transcription factors expression in tissue from IPF

**Fig. 5:**
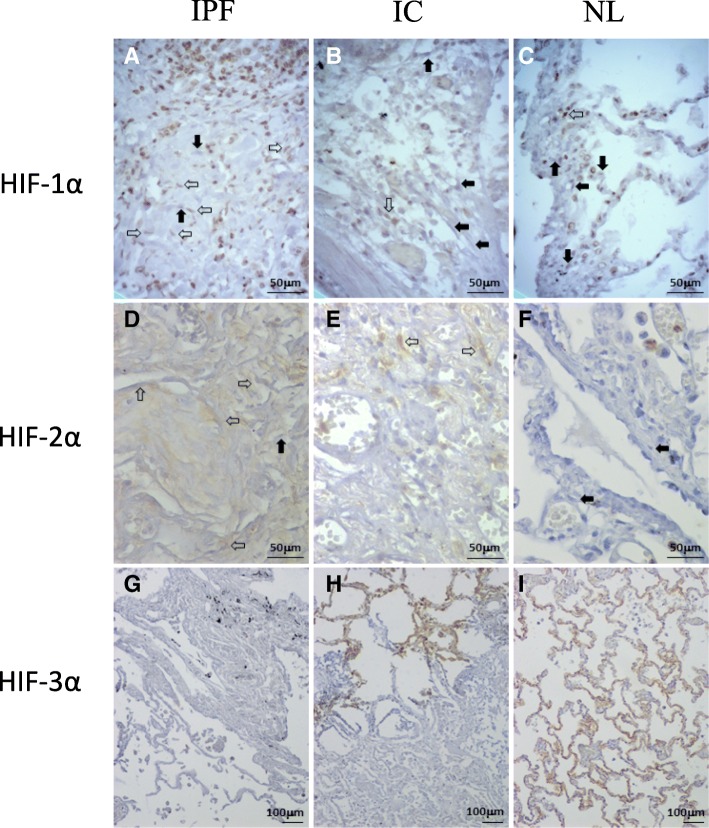
Expression of hypoxia-inducible alpha subunits in normal and diseased lung tissue. HIF-1α and HIF-2α expression are more evident in fibroblasts from idiopathic pulmonary fibrosis (**a**, **d**) than from lung tissue affected by other inflammatory conditions (i.e. chronic bronchitis, panels **b** and **e**) or normal lung tissue (**c**, **f**); as demonstrated by a higher proportion of positive fibroblasts (open arrows) than negative ones (solid black arrows). For HIF-3α expression there are no significant differences in fibroblasts but in the alveolar wall, the latter shows a positive IHC reaction in different cellular elements in controls (**i**) and hypersensitivity pneumonitis (**h**), while no expression in idiopathic pulmonary fibrosis (**g**)

For HIF-1α expression in tissue samples, no differences were encountered in fibroblasts; however, there is a differential distribution of positive cell percentage in idiopathic fibrosis cases in comparison to control lung tissues. Though fibroblasts in tissue samples from both idiopathic fibrosis cases and normal control tissues express HIF-1α, a greater number of idiopathic fibrosis cases have medium or high percentage of positive cells (Fig. 5a, c). From the ranges of positive cells employed for the statistical analysis, the 10–50% range comprises cases with relatively low and medium percentage of positive cells. Setting different thresholds to stratify cases that separate this range at 25% percent, it emphasizes the differential distribution of HIF-1α positive fibroblasts and reflects a trend towards a greater expression of this protein among idiopathic fibrosis cases (*p* = 0.06) (Fig. 5a).

An evident increase in HIF-2α protein expression in fibroblasts from tissue samples was observed in idiopathic fibrosis in comparison to normal control tissue (*p* = 0.0011) (Fig. 5d, f). However, a trend towards an increase in HIF-2α expression was also encountered when comparing tissue samples from other pulmonary inflammatory conditions to normal control tissue (*p* = 0.0878) (Fig. 5e, f). Further, for tisular fibroblasts, there was no difference in expression between idiopathic fibrosis and other inflammatory conditions (*p* = 0.335) (Fig. 5d, e). Significant differences in the expression of this factor were also observed in some inflammatory cells in both subsets of disease when compared to controls: higher percentage of positive plasma cells in idiopathic fibrosis tissues vs normal control (*p* = 0.0429), and higher percentage of positive plasma cells in other inflammatory lung conditions vs normal control (*p* = 0.0242).

For HIF-3α the only significant differences in expression were observed in the septum wall. There was high expression in controls and loss of the stain in the remaining non-lesional lung tissue in idiopathic fibrosis cases (*p* = 0.0151) (Fig. 5g, h, i). This difference remained statistically significant between idiopathic fibrosis cases vs other inflammatory conditions (*p* = 0.0470) (Fig. 5g, h). In contrast to HIF-2α expression for HIF-3α there were no differences in fibroblasts or inflammatory cells, but there was a trend towards lower expression in the epithelial cells alone when comparing their HIF-3α expression between controls and idiopathic fibrosis cases (hyperplastic epithelial cells within the lesion) (*p* = 0.0688) (Fig. 5g, i).

## Discussion

Reduced oxygen tension is observed in many lung disorders, in the context of IPF is considered as a driving force in the progression of the disease. Above all, hypoxic conditions are getting involved in fibroblast foci formation that are the distinctive histopathological feature of this disease. The main objective of this work was to investigate the possible role of hypoxia-regulated transcription factors (HIFs) in fibroblast activation of idiopathic pulmonary fibrosis and age-related controls. On this paper, the analysis of all the factors involved by Westen blot, PCR, IHC and ICC were integrated. Previous studies have already reported that hypoxia promotes myofibroblast differentiation, in primary human AEC cells it induces the expression of αSMA and decreases the expression of E-cadherin [24]. In fibroblasts, this differentiation is through a MMP2-mediated pathway [18, 25, 26]. These results reinforce previous findings, HIF-1α and HIF-2α accumulation contributes to myofibroblast differentiation, as demonstrated in the increased αSMA protein expression in the control group after hypoxia. In the context of IPF, hypoxia signaling is still present in spite of having available oxygen. This indicates that IPF derived fibroblasts are probably working with anaerobic metabolism. There have been found alterations in metabolic pathways related to energetic metabolism in lungs with IPF [27]. This is also supported by other reports that describe a high production of lactate and it leads to assume that these fibroblasts probably have a similar metabolism such as those of cancer [28].

An interesting finding is the involvement of HIF-2α observed at all levels, mRNA and protein expression; in higher magnitude than that of HIF-1α. In congruency with this idea, Lin Q. et al. propose that HIF-1α plays an important role in response to acute hypoxia, while HIF-2α does so against chronic hypoxia [29]. HIF-1α and HIF-2α isoforms show differences in the subset of genes they activate, an example of this could be in the work of Hanna C. et al. in which they observed that HIF-2α plays a significant role in the expression of collagen in human mesangial cells in conditions of normoxia, so they hypothesize that HIF-2α is more important than HIF-1α in normoxic glomerular fibrogenesis [30]. Furthermore, hypoxia potentially stimulates miR-210 expression via HIF-2α, and that high miR-210 expression in turn drives fibroblasts proliferation [9]. All these data correlate with results of the study showing that the expression of HIF-2α is higher and indicate the phenotype seems to be constitutively present in IPF fibroblasts.

Regarding to the HIF-3α isoform, in this work, it was observed that HIF-3α is present in controls, whereas in IPF it is diminished. These finding match with the data of transcriptomic that show lower expression of HIF3α in IPF lungs [31]. HIF-3α is considered a primary factor in alveolarization during lung development, an example of the potent inhibitory effect it has on HIF1α and HIF-2α is that overexpressing mice show a post-pseudoglandular branching defect with a reduced number of airspaces and a clear reduction in the number of alveolar type I and type II cells [32]. This overexpression recapitulates the loss of these factors in lung maturation in newborn.

Due to the fact that there are different transcripts associated with this gene, expression levels of these variants depend on multiple conditions, they can vary depending on the stage of development, for example: HIF3α4 and HIF3α7 are expressed in adult tissues and HIF3α4 in fetal tissue [13, 33]. The expression of this gene by qPCR is not found in the results of this study, while by western blot the polyclonal antibody is able to recognize them, which it can explain this phenomenon at least in part. In lungs of patients with IPF, no HIF-3α expression was observed, maybe due to the low amount of the protein in conjunction with the abundance of collagen and sparsity of fibroblasts. It was observed a high expression of this factor in the septum wall of healthy lung tissue by immunohistochemistry, however, little is known about this protein and much less in adult human lung.

In the same vein, the transcription factors induced by hypoxia have a central role in the distinct phenotype that manifests fibroblast derived from IPF and show an altered response to stress [34, 35]. Therefore, it is propose that αSMA gene expression, at least in part, is determined by the activity of HIF 1α and 2α, in IPF, this increase is greater perhaps due to the lack of regulation of HIF3α. In IPF, hypoxia hypermethylation could be one of the conditions by which the pathways involved in the response to stress could be altered. One of the limitations is the lack of significance difference in the IPF lines treated with the demethylating agent, because this treatment partially recovers HIF3α expression in IPF derived fibroblast. So probably some other mechanisms are involved in the reduction of HIF-3α which needs to be investigated more thoroughly.

## Conclusions

Our data, altogether, reinforce the idea that hypoxia is a determining factor in the development and progression of fibrosis. We have showed a correlation between overactivation in myofibroblast differentiation as a result of the increase in HIF-1α and HIF-2α. Also, it is noted that IPF fibroblasts have a decrease in HIF-3α which could be related to its hypermethylation and this means a possible mechanism of susceptibility in patients with IPF.

## Data Availability

Not applicable.

## Abbreviations

5-Aza: 5-Aza-2′-deoxycytidine
BCA: Bicinchoninic acid
ECM: Extracellular matrix
HIF: Hypoxia inducible factor
HRE: Hypoxia response elements
ICC: Immunocytochemistry
IHC: Immunohistochemistry
IPF: Idiopathic pulmonary fibrosis
MMP2: Matrix Metalloproteinase-2
PAGE: Polyacrylamide gels
PBS: Phosphate-buffered saline
TMA: Tissue microarray
VHL: Von Hippel–Lindau
αSMA: Alpha smooth muscle actin

## References

[1] Selman M, King TE, Pardo A (2001). Idiopathic pulmonary fibrosis: prevailing and evolving hypotheses about its pathogenesis and implications for therapy. Ann Intern Med.

[2] King TE, Pardo A, Selman M (2011). Idiopathic pulmonary fibrosis. Lancet..

[3] Selman M, Pardo A (2014). Revealing the pathogenic and aging-related mechanisms of the enigmatic idiopathic pulmonary fibrosis. An integral model. Am J Respir Crit Care Med.

[4] Shimoda LA, Semenza GL (2011). HIF and the lung: role of hypoxia-inducible factors in pulmonary development and disease. Am J Respir Crit Care Med.

[5] Semenza GL (1999). Regulation of mammalian O_2_ homeostasis by hypoxia-inducible factor 1. Annu Rev Cell Dev Biol.

[6] Tzouvelekis A, Harokopos V, Paparountas T, Oikonomou N, Chatziioannou A, Vilaras G (2007). Comparative expression profiling in pulmonary fibrosis suggests a role of hypoxia-inducible factor-1alpha in disease pathogenesis. Am J Respir Crit Care Med.

[7] Higgins DF, Kimura K, Bernhardt WM, Shrimanker N, Akai Y, Hohenstein B (2007). Hypoxia promotes fibrogenesis in vivo via HIF-1 stimulation of epithelial-to-mesenchymal transition. J Clin Invest.

[8] Moon J-O, Welch TP, Gonzalez FJ, Copple BL (2009). Reduced liver fibrosis in hypoxia-inducible factor-1alpha-deficient mice. Am J Physiol Gastrointest Liver Physiol.

[9] Bodempudi V, Hergert P, Smith K, Xia H, Herrera J, Peterson M (2014). miR-210 promotes IPF fibroblast proliferation in response to hypoxia. AJP Lung Cell Mol Physiol.

[10] Senavirathna LK, Huang C, Yang X, Munteanu MC, Sathiaseelan R, Xu D (2018). Hypoxia induces pulmonary fibroblast proliferation through NFAT signaling. Sci Rep.

[11] Raghu G, Collard HR, Egan JJ, Martinez FJ, Behr J, Brown KK (2011). An official ATS/ERS/JRS/ALAT statement: idiopathic pulmonary fibrosis: evidence-based guidelines for diagnosis and management. Am J Respir Crit Care Med.

[12] Raghu G, Remy-Jardin M, Myers JL, Richeldi L, Ryerson CJ, Lederer DJ (2018). Diagnosis of idiopathic pulmonary fibrosis. Am J Respir Crit Care Med.

[13] Augstein A, Poitz DM, Braun-Dullaeus RC, Strasser RHSA (2011). Cell-specific and hypoxia-dependent regulation of human HIF-3α: inhibition of the expression of HIF target genes in vascular cells. Cell Mol Life Sci.

[14] Heikkilä M, Pasanen A, Kivirikko KI, Myllyharju J (2011). Roles of the human hypoxia-inducible factor (HIF)-3a variants in the hypoxia response. Cell Mol Life Sci.

[15] Yang SL, Wu C, Xiong ZF, Fang X (2015). Progress on hypoxia-inducible factor-3: its structure, gene regulation and biological function (review). Mol Med Rep.

[16] Heidbreder M, Fröhlich F, Jöhren O, Dendorfer A, Qadri F, Dominiak P (2003). Hypoxia rapidly activates HIF-3alpha mRNA expression. FASEB J.

[17] Tanaka T, Wiesener M (2009). Wanja Bernhardt K-UE and CW. The human HIF (hypoxia-inducible factor)-3α gene is a HIF-1 target gene and may modulate hypoxic gene induction. Biochem J.

[18] Robinson CM, Neary R, Levendale A, Watson CJ, Baugh JA (2012). Hypoxia-induced DNA hypermethylation in human pulmonary fibroblasts is associated with Thy-1 promoter methylation and the development of a pro-fibrotic phenotype. Respir Res.

[19] Ravenna L, Salvatori L, Russo MA (2016). HIF3α: the little we know. FEBS J.

[20] Maynard MA, Qi H, Chung J, Lee EHL, Kondo Y, Hara S (2003). Multiple splice variants of the human HIF-3α locus are targets of the von Hippel-Lindau E3 ubiquitin ligase complex. J Biol Chem.

[21] Pasanen A, Heikkilä M, Rautavuoma K, Hirsilä M, Kivirikko KI, Myllyharju J (2010). Hypoxia-inducible factor (HIF)-3α is subject to extensive alternative splicing in human tissues and cancer cells and is regulated by HIF-1 but not HIF-2. Int J Biochem Cell Biol.

[22] Rabinovich EI, Kapetanaki MG, Steinfeld I, Gibson KF, Pandit KV, Yu G (2012). Global methylation patterns in idiopathic pulmonary fibrosis. PLoS One.

[23] Geybels MS, Zhao S, Wong C, Bibikova M, Wu M, Ostrander EA (2016). Epigenomic profiling of DNA methylation in paired prostate cancer versus adjacent benign tissue. Prostate..

[24] Zhou G, L a D, Wu M, Kelly A, Trejo H, Zhou Q (2009). Hypoxia-induced alveolar epithelial-mesenchymal transition requires mitochondrial ROS and hypoxia-inducible factor 1. Am J Physiol Lung Cell Mol Physiol.

[25] Tomasek JJ, Gabbiani G, Hinz B, Chaponnier C (2002). Brown R a. Myofibroblasts and mechano-regulation of connective tissue remodelling. Nat Rev Mol Cell Biol.

[26] Misra S, Fu AA, Misra KD, Shergill UM, Leof EB, Mukhopadhyay D (2010). Hypoxia-induced phenotypic switch of fibroblasts to myofibroblasts through a matrix metalloproteinase 2/tissue inhibitor of metalloproteinase-mediated pathway: implications for venous neointimal hyperplasia in hemodialysis access. J Vasc Interv Radiol.

[27] Zhao YD, Yin L, Archer S, Lu C, Zhao G, Yao Y (2017). Metabolic heterogeneity of idiopathic pulmonary fibrosis: A metabolomic study. BMJ Open Respir Res.

[28] Kottmann RM, A a K, K a S, Lyda E, Dahanayake T, Salibi R (2012). Lactic acid is elevated in idiopathic pulmonary fibrosis and induces myofibroblast differentiation via pH-dependent activation of transforming growth factor-β. Am J Respir Crit Care Med.

[29] Lin Q, Cong X, Yun Z (2011). Differential hypoxic regulation of hypoxia-inducible factors 1alpha and 2alpha. Mol Cancer Res.

[30] Hanna C, Hubchak SC, Liang X, Rozen-Zvi B, Schumacker PT, Hayashida T (2013). Hypoxia-inducible factor-2α and TGF-β signaling interact to promote normoxic glomerular fibrogenesis. Am J Physiol Renal Physiol.

[31] Gangwar I, Kumar Sharma N, Panzade G, Awasthi S, Agrawal A, Shankar R (2017). Detecting the molecular system signatures of idiopathic pulmonary fibrosis through integrated genomic analysis. Sci Rep.

[32] Huang Yadi, Kapere Ochieng Joshua, Kempen Marjon Buscop-van, Munck Anne Boerema-de, Swagemakers Sigrid, van IJcken Wilfred, Grosveld Frank, Tibboel Dick, Rottier Robbert J. (2013). Hypoxia Inducible Factor 3α Plays a Critical Role in Alveolarization and Distal Epithelial Cell Differentiation during Mouse Lung Development. PLoS ONE.

[33] Yamashita T, Ohneda O, Nagano M, Iemitsu M, Makino Y, Tanaka H (2008). Abnormal heart development and lung remodeling in mice lacking the hypoxia-inducible factor-related basic helix-loop-helix PAS protein NEPAS. Mol Cell Biol.

[34] Ramos C, Montaño M, García-Alvarez J, Ruiz V, Uhal BD, Selman M (2001). Fibroblasts from idiopathic pulmonary fibrosis and normal lungs differ in growth rate, apoptosis, and tissue inhibitor of metalloproteinases expression. Am J Respir Cell Mol Biol.

[35] Romero Y, Bueno M, Ramirez R, Álvarez D, Sembrat JC, Goncharova EA, Rojas M, Selman M, Mora AL, Pardo A (2016). mTORC1 activation decreases autophagy in aging and idiopathic pulmonary fibrosis and contributes to apoptosis resistance in IPF fibroblasts. Aging Cell.

